# Endoscopic thyroidectomy via areola approach for stage T1 papillary thyroid carcinoma: feasibility, safety, and oncologic outcomes

**DOI:** 10.3389/fendo.2023.1212490

**Published:** 2023-11-23

**Authors:** Jinlong Huo, Yaxuan Xu, Jie Yu, Youming Guo, Xiaochi Hu, Dong Ou, Rui Qu, Lijin Zhao

**Affiliations:** ^1^ Department of General Surgery, Digestive Disease Hospital, Affiliated Hospital of Zunyi Medical University, Zunyi, Guizhou, China; ^2^ Department of Breast and Thyroid Surgery, The Third Affiliated Hospital of Zunyi Medical University (The First People’s Hospital of Zunyi), Zunyi, Guizhou, China; ^3^ Department of Oncology, The Third Affiliated Hospital of Zunyi Medical University (The First People’s Hospital of Zunyi), Zunyi, Guizhou, China

**Keywords:** papillary thyroid carcinoma, endoscopic thyroidectomy, areola approach, operative safety, quality of life

## Abstract

**Purpose:**

To evaluate the feasibility, safety, and oncologic outcomes associated with endoscopic thyroidectomy via the areolar approach (ETAA), compared with conventional open thyroidectomy (COT) for the treatment of stage T1 papillary thyroid carcinoma (PTC).

**Methods:**

Between January 2021 and June 2022, a total of 1204 patients diagnosed with PTC underwent screening, out of which 138 patients were selected for inclusion in the study population after propensity score matching (92 patients in the ETAA group and 46 patients in the COT group). The study included the collection and analysis of clinicopathologic characteristics, intraoperative outcomes, postoperative outcomes, complications, and follow-up data using R software.

**Results:**

The operative time for the ETAA group was longer than that for the COT group (160.42 ± 32.21 min vs. 121.93 ± 29.78 min, p < 0.0001). However, there were no significant differences between the two groups in terms of intraoperative blood loss, the extent of surgical resection, the number of dissected lymph nodes, the number of metastatic lymph nodes, and the rate of parathyroid autotransplantation. Postoperative drainage and C-reactive protein levels were higher in the ETAA group than in the COT group, but there were no significant differences in 24-hour visual analogue scale scores, white blood cell counts, drainage duration, or postoperative hospital stay. Complication rates were similar between the two groups, and no permanent recurrent laryngeal nerve palsy or hypoparathyroidism was observed. Patients who underwent ETAA reported greater cosmetic satisfaction and quality of life than those who underwent COT. During the follow-up phase, only one patient in the COT group developed lateral cervical lymph node involvement requiring reoperation.

**Conclusion:**

ETAA is a safe and feasible surgical method for patients with stage T1 PTC, providing results similar to COT in terms of oncologic completeness, while avoiding neck scars, with excellent cosmetic effects.

**Clinical trial registration:**

Chinese Clinical Trial Registry center, identifier ChiCTR2300077109

## Introduction

Papillary thyroid carcinoma (PTC) is the most prevalent form of thyroid cancer, representing roughly 80% of all thyroid malignancies (1, 2). Surgical resection continues to be the principal therapy for PTC. Conventional open thyroidectomy(COT) has traditionally been viewed as the preferred method to treat PTC, requiring a sizable incision in the neck. However, due to cosmetic advantages, scarless endoscopic thyroidectomy has gained favour in recent years with advances in surgical techniques.

Currently, surgeons have developed various techniques for endoscopic thyroid surgery techniques, including the transoral vestibular (3–5), trans-axillary (6), anterior breast (7), and transcervical approaches (8), as well as hybrid approaches such as the axillary bilateral breast approach (9), bilateral axillary-breast approach (10), and unilateral trans-axillary-trans-mammary approach (11). Each approach has its distinct characteristics, advantages, and drawbacks. In China, Endoscopic Thyroidectomy via the Areola Approach(ETAA) has emerged as an alternative technique to COT for treating PTC. This technique involves making a small incision around the areola for access to the thyroid gland, reducing the visible scarring on the neck. By utilizing the endoscopic approach, surgeons can visualize and manipulate the thyroid gland and surrounding lymph nodes, with improved precision and minimal tissue disruption.

For the diagnosis and treatment of differentiated thyroid cancer, the Chinese guidelines recommend that patients with tumors smaller than 1 cm are advised to undergo lobectomy and prophylactic central lymph node dissection (CLND) (12, 13). However, there are several concerns regarding the use of ETAA for treating PTC. One of the primary concerns is the difficulty in entirely removing central neck lymph nodes due to the inferior-to-superior approach of the ETAA view and the obstruction caused by the clavicle (7, 14, 15). Advancements in surgical skills enabled ETAA to demonstrate comparable oncological results and surgical outcomes to COT for PTC smaller than 1 cm in size, as reported in several studies (16–20). Nonetheless, few comprehensive studies have reported on the feasibility, safety, and oncologic outcomes of ETAA for Stage T1 PTC.

The objective of this clinical study is to assess the feasibility, safety, and cancer-related results of ETAA in patients diagnosed with stage T1 PTC. This contribution to the current literature on minimally invasive thyroid surgery may offer additional evidence for the appropriateness of utilizing the areola approach in treating PTC. Ultimately, this study aims to strengthen the use of ETAA as a secure and efficient treatment alternative for patients diagnosed with stage T1 PTC, further advancing the field of thyroid surgery.

## Materials and methods

### Patients

A retrospective analysis was conducted on a cohort of 1,204 patients diagnosed with PTC who underwent either ETAA or COT at the Third Affiliated Hospital of Zunyi Medical University (The First People’s Hospital of Zunyi) between January 2021 and June 2022. Preoperative imaging assessments, such as ultrasound and computed tomography, were performed to evaluate the tumour and lymph node status.

The exclusion criteria for participants in this study included the following factors (1): Age less than 18 or exceeding 60 years (2); Pre-operative ultrasound indicating a tumour size exceeding 2cm (3); Distant metastasis or direct invasion of the surrounding organs was confirmed by preoperative imaging (4); Abnormal cervical lymph nodes observed and/or distant metastasis; (5) History of prior neck surgery or radiation therapy; (6) Poor general condition and inability to tolerate general anaesthesia; and (7) other histologic type cancers. This study received approval from the Ethics Committee of the First People’s Hospital of Zunyi and ensured that all patients provided written informed consent. The analysis enrolled patients with PTC measuring 2cm or less in diameter, classified as Stage T1 according to the 8th edition AJCC staging guidelines, and exhibiting negative margins (21). The study has been registered in the Chinese Clinical Trial Registry center (No. ChiCTR2300077109).

### Propensity score matching analysis

To reduce selection bias, we carried out propensity score matching (PSM) (22, 23). We generated propensity scores for individuals using logistic regression analysis performed with SPSS software (version 26.0; IBM Corp, Armonk, NY, USA). For PSM matching, we chose seven clinicopathological factors as covariates that might have an impact on surgical outcomes. These factors included age, gender, tumour size, invasion of the thyroid capsule, multifocality of tumors in the same lobe, tumour location, and the presence of Hashimoto’s thyroiditis. After considering these variables, we utilized a 1:2 PSM technique using the nearest neighbour method, with a calliper width set at 0.06 standard deviations of the logit of the propensity score (22, 23).

### Surgical procedures

**Figure 1:**
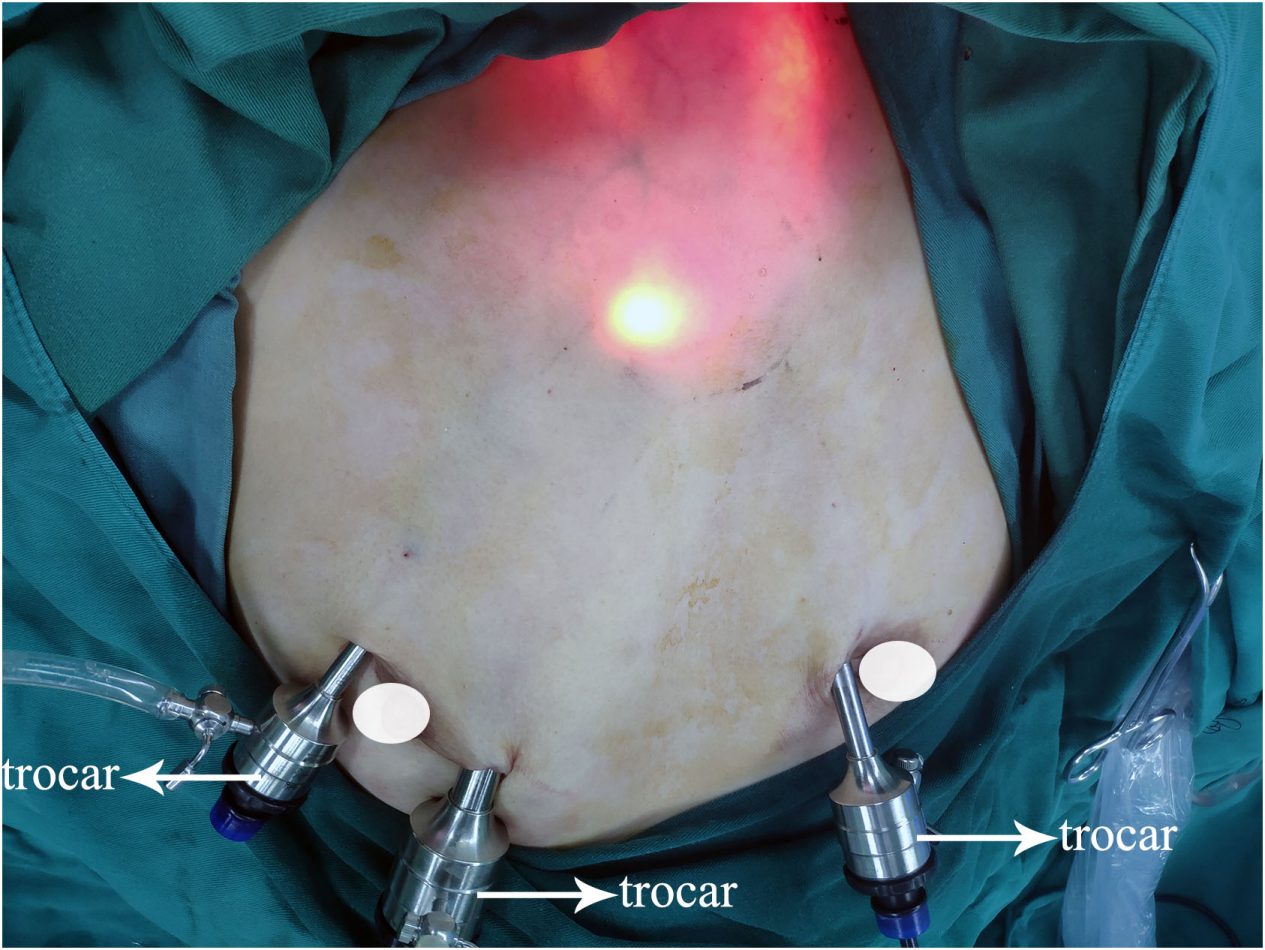
Trocar placement.

**Figure 2:**
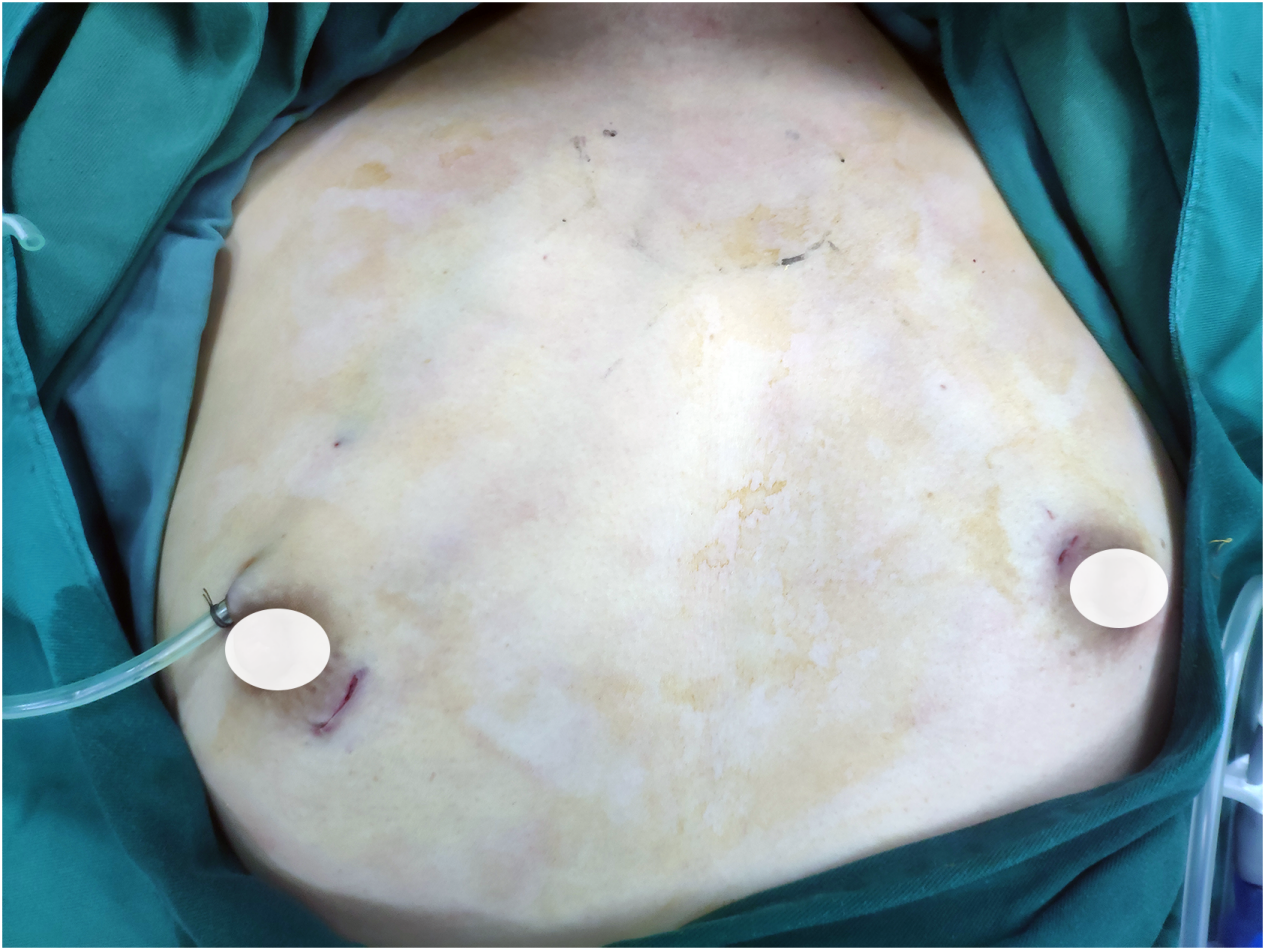
The incisions of ETAA in female patients. ETAA, endoscopic thyroidectomy via the areola approach;.

Surgical strategies were carried out as per the 2012 Chinese guidelines (24), which recommended hemithyroidectomy or total thyroidectomy along with prophylactic CLND to patients diagnosed with PTC. Operative procedures for patients with PTC have been described previously by ETAA and COT (7, 20, 25). Furthermore, we have refined the steps of ETAA to improve the surgical feasibility based on our center’s accumulated experience. For instance, a four-step protocol for CLND has been introduced. The procedure for dissection involves four sequential steps (1): dissection of the anterior lymphatic and adipose tissues of the larynx, (2) dissection of the lower central zone tissue along the anterior cervical muscle group, (3) exposure of the common carotid artery, and (4) separation and removal of the central lymph nodes along the recurrent laryngeal nerve. For specific details on all ETAA procedures, refer to the attached **Supplementary Material**. **Figure 1** illustrates the placement of the trocar during ETAA, while **Figure 2** demonstrates the body surface incision performed after surgery in female patients. Our steps for handling thyroidectomy under COT are similar to those for ETAA.

### Perioperative management and follow-up

Patient data was collected and classified into four groups: clinical pathological characteristics including age, gender, body mass index (BMI), tumour size, multifocality status, tumour location, and the presence of Hashimoto’s thyroiditis. Ultrasound was used to assess tumour size, and the maximum diameter was recorded. Multifocality was defined as having two or more lesions. Intraoperative outcomes were evaluated, including operative time, intraoperative blood loss, the extent of surgical resection, number of dissected lymph nodes, number of metastatic lymph nodes, rate of signal attenuation in the recurrent laryngeal nerve, rate of parathyroid autotransplantation, and incidences of conversion to an open procedure during surgery. The study evaluated postoperative results, which included assessment of the 24-hour visual analogue scale (VAS), white blood cell count, C-reactive protein level, total drainage volume, drainage duration, length of postoperative hospital stay, and occurrence of postoperative complications. Additionally, the follow-up assessment encompassed self-reported scar awareness, satisfaction with the cosmetic outcome, quality of life, oncologic completeness, and recurrence.

In terms of complications, the literature defines vocal cord palsy as transient if it occurs within 2 weeks of surgery, and permanent if present beyond 6 months post-surgery (26). Transient hypoparathyroidism is indicated by a post-surgical iPTH level below the normal range (15 ng/l). Postoperative hypoparathyroidism is characterized by persistent hypocalcemia with consistently low parathyroid hormone levels (10 pg/mL) for over 1 year after surgery (27).

According to the follow-up recommendations outlined by the American Thyroid Association(ATA), it is advised that all patients receive regular monitoring for recurrence via serum thyroid function tests and ultrasound (28). These assessments should be conducted every 3-6 months to closely observe any possible indications of recurrence. Patients who have undergone total thyroidectomy should have their serum-stimulated thyroglobulin (sTg) level assessed and their percentage of sTg below 1 mg/L measured three weeks after discharge. Based on the results, radioactive iodine therapy may be recommended as a treatment option. In addition, a survey evaluating cosmetic satisfaction, scar self-awareness, and quality of life was administered to patients three months after surgery. Cosmetic satisfaction and scar self-awareness were evaluated using a scoring system ranging from 0 to 3, with higher scores indicating higher satisfaction. Quality of life scores were measured on a scale from 0 (worst) to 10 (best).

### Statistical analysis

The data analysis was performed using R software (version 3.5.3; R core team, Vienna, Austria). The mean ± standard deviation was used to present continuous variables, while percentages were used for categorical variables. Students’ t-tests were conducted for continuous data, and Chi-square, Fisher’s exact, or Mann-Whitney U tests for categorical data. Statistical significance was established when the p-value (p) was less than 0.05.

## Results

### Clinicopathologic characteristics after PSM

**Figure 3:**
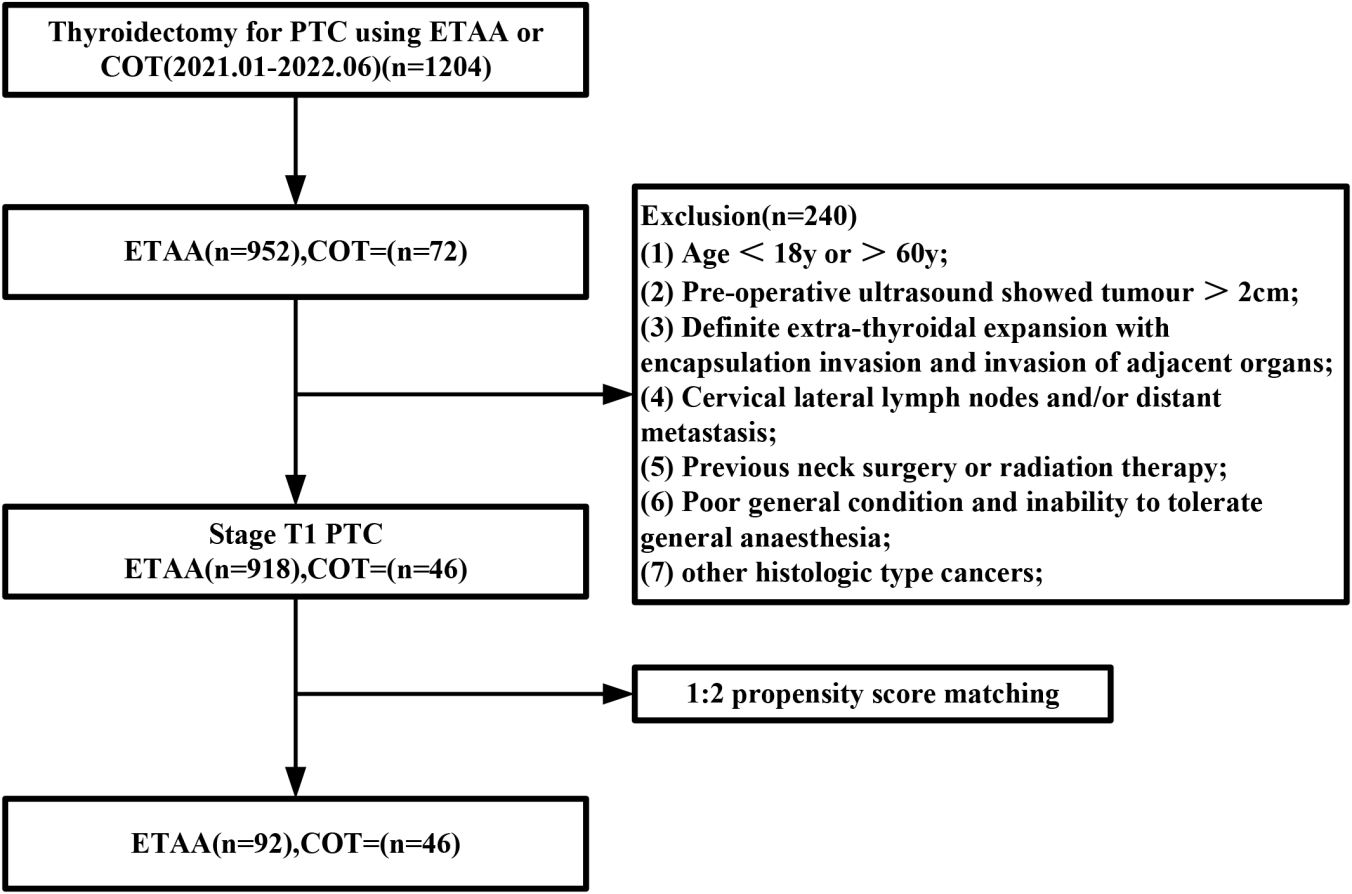
Flowchart of propensity score-matching. ETAA, endoscopic thyroidectomy via the areola approach; COT, conventional open thyroidectomy.

A total of 964 patients were eligible for inclusion in the study. Of these, 918 were part of the ETAA group, and the remaining 46 patients were assigned to the COT group. After conducting PSM, the study size decreased to 138 patients (n=92 in the ETAA group, n=46 in the COT group), as shown in **Figure 3**. The two matched groups displayed a well-balanced distribution of the seven covariates after PSM. No significant differences were observed in the clinicopathological characteristics between the two groups, as shown in **Table 1**.

**Table 1:** Comparison of clinicopathological characteristics after propensity score matching.

Variables	ETAA (n = 92)	COT(n=46)	*P*
Age ( years, mean ± SD)	32.18 ± 7.62	34.30 ± 7.44	0.123
Gender(n, %)			1.000
Male	16 (17.4)	8 (17.4)	
Female	76 (82.6)	38 (82.6)	
BMI (kg/m2, mean ± SD)	22.25 ± 2.85	22.32 ± 2.46	0.887
Diameter of the largest tumor(cm, mean ± SD)	0.85 ± 0.46	0.88 ± 0.59	0.764
Thyroid capsule invasion			0.807
Yes	38 (41.3)	20 (43.5)	
No	54 (58.7)	26 (56.5)	
Tumor multifocality in the same lobe			
Yes	52 (56.5)	26 (56.5)	1.000
No	40 (43.5)	20 (43.5)	
Tumor location			0.603
Left	39 (42.4)	17 (36.9)	
Isthmus	3 (3.3)	1 (2.2)	
Right	30 (32.6)	16 (34.8)	
Both	20 (21.7)	12 (26.1)	
Hashimoto’s thyroiditis (n, %)			0.623
Yes	40 (43.5)	18 (39.1)	
No	52 (56.5)	28 (60.9)	

BMI, body mass index; ETAA, endoscopic thyroidectomy via areola approach; COT, conventional open thyroidectomy.

### Intraoperative outcomes

Operation time was significantly longer in the ETAA group than in the COT group(160.42 ± 32.21 min vs. 121.93 ± 29.78 min,p < 0.0001). However, no statistically significant differences existed between the two groups concerning blood loss, the number of dissected lymph nodes, positive lymph nodes, and the results of parathyroid autotransplantation. Not a single instance case in the ETAA group necessitated a conversion to the COT. Further information about both groups can be found in **Table 2**.

**Table 2:** Comparison of intraoperative outcomes.

Variables	ETAA (n = 92)	COT(n=46)	*P*
Operative time (min, mean ± SD)	160.42 ± 32.21	121.93 ± 29.78	<0.0001
Blood loss (ml, mean ± SD)	21.09 ± 11.21	19.72 ± 10.62	0.492
Extent of surgery (n, %)			0.145
Lobectomy	69 (75.0)	29 (63.1)	
Total thyroidectomy	23 (25.0)	17 (36.9)	
Number of dissected lymph nodes (piece)			0.808
Positive (n, %)	40 (43.5)	19 (41.3)	
Negative (n, %)	52 (56.5)	27 (58.7)	
Number of positive lymph nodes (piece, mean ± SD)	2.96 ± 2.23	3.42 ± 2.34	0.263
Rate of parathyroid autotransplantation (n, %)	42 (45.7)	22 (47.8)	0.809
Conversion to open	0	NA	NA

ETAA, endoscopic thyroidectomy via areola approach; COT, conventional open thyroidectomy.

### Postoperative outcomes and complications

**Table 3** presents the postoperative outcomes and complications. The ETAA group showed higher levels of C-reactive protein in patients 24 hours after surgery (8.94 ± 6.97 mg/L vs 6.32 ± 4.54 mg/L, p < 0.009) and a higher volume of total drainage (162.43 ± 68.92 ml vs 120.51 ± 76.48 ml, p = 0.001) than the COT group. However, there were no significant differences were observed between the two groups in terms of 24-hour VAS scores (1.69 ± 0.54 vs 1.72 ± 0.48, p = 0.75), WBC counts (11.17 ± 2.71 × 10^9^/L vs 10.18 ± 5.23 × 10^9^/L, p = 0.233), duration of drainage (3.15 ± 1.22 days vs 3.54 ± 1.04 days, p = 0.066), and postoperative hospital stay (3.25 ± 1.26 days vs 3.59 ± 1.31 days, p = 0.143).

**Table 3:** Comparison of postoperative outcomes and complication.

Variables	ETAA (n = 92)	COT(n=46)	*P*
24-h VAS (score, mean ± SD)	1.69 ± 0.54	1.72 ± 0.48	0.75
WBC amount (10^9^/L, mean ± SD)	11.17 ± 2.71	10.18 ± 5.23	0.233
C-reactive protein (mg/L, mean ± SD)	8.94 ± 6.97	6.32 ± 4.54	**0.009**
Duration of drainage (days, mean ± SD)	3.15 ± 1.22	3.54 ± 1.04	0.066
Total drainage amount (ml, mean ± SD)	162.43 ± 68.92	120.51 ± 76.48	**0.001**
Postoperative hospital stay (days, mean ± SD)	3.25 ± 1.26	3.59 ± 1.31	0.143
Complication (n,%)			
Hypoparathyroidism			
Transient	21 (22.8)	11 (23.9)	0.887
Permanent	0 (0)	0 (0)	NA
Vocal cord palsy			
Transient	24 (26.1)	9 (19.6)	0.397
Permanent	0 (0)	0 (0)	NA
Chylous fistula	1 (1.0)	2 (4.3)	0.258
Seroma	6 (6.5)	4 (8.6)	0.731
Bleeding	0 (0)	0 (0)	NA
Infection	0 (0)	0 (0)	NA

VAS, visual analog scale; WBC, white blood cells; ETAA, endoscopic thyroidectomy via areola approach; COT, conventional open thyroidectomy. Bold values denotes statistically significant difference.

Several complications were observed among the patients, including transient hypoparathyroidism (ETAA: 21/92, COT: 11/46), transient recurrent laryngeal nerve palsy (ETAA: 24/92, COT: 9/46), chylous fistula (ETAA: 1/92, COT: 2/46), and seroma (ETAA: 6/92, COT: 4/46), with no significant differences between the two groups. Neither group had any cases of permanent recurrent laryngeal nerve palsy, hypoparathyroidism, postoperative haemorrhage, or infection.

### Postoperative follow up

All patients were followed up until March 20, 2023, with no statistically significant differences in the mean follow-up time between the two groups (**Table 4**). The ETAA group had higher levels of cosmetic satisfaction (3.26 ± 0.57 vs 2.25 ± 0.61, p < 0.0001), lower self-consciousness about scar (0.74 ± 0.58 vs 2.23 ± 0.46, p < 0.0001), and improved quality of life (8.65 ± 1.02 ml vs 7.76 ± 0.85, p <0.0001) compared to the COT group. Regarding oncologic completeness in total thyroidectomy, there were no statistically significant differences were observed between the two groups in the stimulated thyroglobulin (Tg) level before radioiodine ablation (RAI) (1.21± 2.42 mg/L vs. 1.32 ± 1.89 mg/L, p = 0.77), the proportion of patients with a Tg level <1 mg/L (78.3% vs. 70.6%, p = 0.717) or >1mg/L (21.7% vs. 29.4%, p = 0.717), and rate of the receiving RAI (47.8% vs. 35.3%, p = 0.525). During the follow-up period, a single patient in the COT group experienced recurrence in the lateral cervical area and consequently went through a second operation. There was no significant difference observed between the two groups in terms of recurrence rate(p = 0.333).

**Table 4:** Comparison of follow-up outcomes.

Variables	ETAA (n = 92)	COT(n=46)	*P*
Cosmetic satisfaction (score, mean ± SD)	3.26 ± 0.57	2.25 ± 0.61	**<0.0001**
Scar self-consciousness (score, mean ± SD)	0.74 ± 0.58	2.23 ± 0.46	**<0.0001**
Quality of life (score, mean ± SD)	8.65 ± 1.02	7.76 ± 0.85	**<0.0001**
Oncologic completeness			
In total thyroidectomy			
Stimulated Tg level before RAI (mg/L, mean ± SD)	1.21 ± 2.42	1.32 ± 1.89	0.77
<1 (n, %)	18/23(78.3)	12/17(70.6)	0.717
≥1 (n, %)	5/23(21.7)	5/17(29.4)	0.717
Rate of receiving RAI (n, %)	11/23(47.8)	6/17(35.3)	0.525
Recurrence (n, %)	0(0)	1(2.2)	0.333
Follow-up duration (months, mean ± SD)	17.52 ± 4.25	17.64 ± 4.06	0.768

Tg, thyroglobulin; RAI, radioactive iodine; ETAA, endoscopic thyroidectomy via areola approach; COT, conventional open thyroidectomy. Bold values denotes statistically significant difference.

## Discussion

In this study, we employed ultrasound-guided fine-needle aspiration biopsy to conduct screening for patients diagnosed with PTC, specifically limited to stage T1, at our centre. We utilized PSM to adjust for the demographic characteristics of the populations included in the ETAA and COT groups. Following the screening process, a total of 138 patients were included in a 1:2 ratio and subsequently assigned to either the ETAA group (92 patients) or the COT group (46 patients). Our analysis revealed that the ETAA group exhibited a significantly longer operative time in comparison to the COT group. Furthermore, the ETAA group demonstrated elevated postoperative drainage volume and C-reactive protein levels in contrast to the COT group. Despite the absence of statistical difference in surgical complications between the two groups, the ETAA group exhibited significantly improved postoperative cosmetic scores and quality of life in comparison to the COT group.

Elevated levels of C-reactive protein have a robust correlation with infections (29). The ETAA group showed higher levels of C-reactive protein than the COT group at 24 hours after surgery, likely due to the longer operative time and more extensive flap separation. Despite the higher total drainage volume in the ETAA group, there was no increase in postoperative drainage time or discharge time. In addition, even though the ETAA group had a larger separation area in their flap, there was no routine administration of postoperative analgesia in either group, and no significant difference was observed in VAS scores between the two groups. The incidence of complications related to surgery did not increase in the ETAA group. Additionally, there were no statistically significant differences between the two groups in terms of postoperative haemorrhage and seroma, which are often associated with more extensive flap separation. Therefore, we believe this observed increase in surgical trauma to be acceptable.

**Figure 4:**
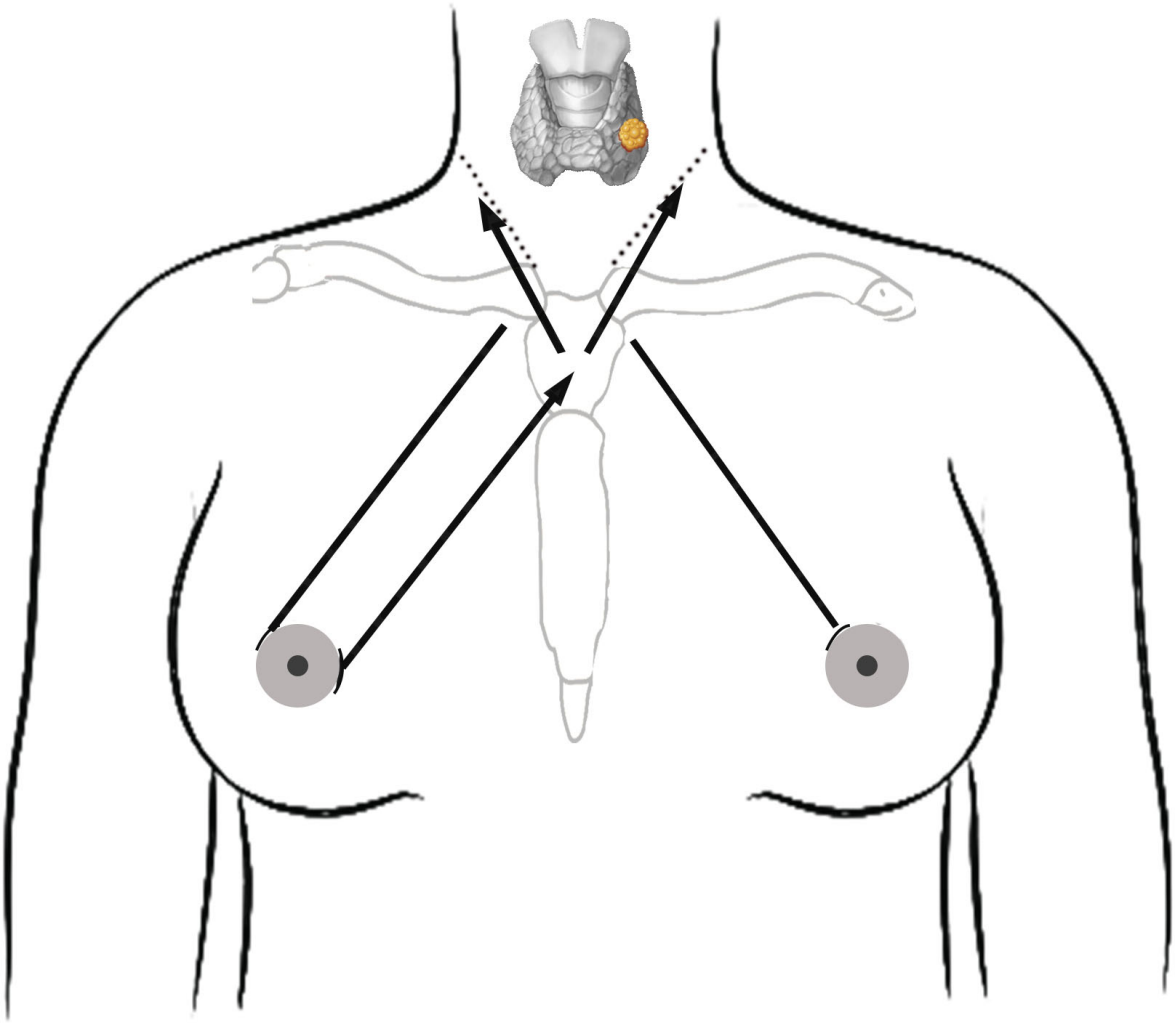
Schematic diagram of the procedure for establishing the surgical cavity. Solid arrow lines indicates the direction of freeing of the blunt separation rod. The dotted line is the lateral border of the sternocleidomastoid muscle.Solid lines on both sides indicate the direction of trocar placement.

The creation of a surgical area is crucial for the successful execution of ETAA procedures. However, inexperienced individuals can face obstacles in this matter. Prior studies suggest that to enhance the surgical process in ETAA, the dissection range should extend to the lateral boundary of the sternocleidomastoid muscle on both sides, the upper edge of the thyroid cartilage, and the clavicle at the lower end (7, 20, 25). Using visualization instruments significantly helps in successful cavity construction, effectively reducing the risk of bleeding. Nonetheless, it has the drawback of increasing the total operative time. In our experience, we have found that non-visualized instruments based on thoracic surface markers can significantly reduce the time required for cavity creation. As body markers (**Figure 4**), we utilized the sternal stalk, bilateral clavicular heads, and bilateral lateral margins of the sternocleidomastoid muscle. Using a syringe, we injected a total volume of 70 ml of tumescent solution(1 ml of epinephrine mixed with 500 ml of fluid) through the right areolar incision up to the top of the sternal stalk. To access the lateral border of the sternocleidomastoid muscle, a blunt dissection instrument was used to free the subcutaneous tissue towards the lateral border of the clavicular heads bilaterally (see **Figure 4**). It is recommended to perform dissection at the adipose layer to avoid damage to the deep internal mammary artery, which could lead to bleeding. Additionally, placement of the primary trocar often leads to a false tract. To address this issue, we utilize larger separator forceps to dilate the lumen before inserting the main trocar. This enables smooth injection through the observation hole and effectively avoids creating a false tract. Some studies have reported non-visualized lumpectomy procedures that involve using an ultrasonic knife for dissecting neck tissue without visualization (30). However, our approach may be more practical. While the procedure time for our non-visualized cavity construction is significantly shorter compared to similar studies, it still exceeds that of COT.

Thyroid lobectomy typically involves the release of the peri-thyroidal tissue to achieve complete exposure of the thyroid gland (25). Throughout the surgery, while adhering to safety standards, the specific surgical steps for resection may vary based on the surgeon’s preference. At our center, we usually begin the procedure by performing isthmus dissection, followed by lateral lobe dissection. This approach enhances mobility on one side of the focal lobe, making gland retraction and excision easier thereafter.

Protecting the recurrent laryngeal nerve(RLN) from injury is paramount during various thyroid-related procedures (31, 32). Although understanding its anatomy is fundamental, the utilization of nerve monitoring has been shown to be effective in reducing the risk of RLN injury (33, 34). In our study, we consistently used nerve monitoring to localize the RLN in both groups, thus avoiding injury to the RLN. In addition, the magnification effect of the lumpectomy may have additionally contributed to the prevention of RLN injury in the ETAA group. Consistent with previous reports, the incidence rate of transient RLN injury varies from 1% to 25% (35, 36), which is similar to our results(23.9%). Improper intraoperative nerve traction and heat exposure may also lead to transient RLN paralysis. To mitigate the risk of transient RLN palsy during surgery, we take precautions including gentle tissue retraction, the use of ultrasound knives in low-power mode while dissociating tissue adjacent to the RLN, and judicious use of continuous excitation to prevent overheating. Occasionally, We utilize gauze to isolate the RLN during surgery to prevent heat-induced damage from the ultrasound knife.

Previous reports have shown a wide range in the occurrence rate of transient hypoparathyroidism, ranging from 1.2% to 40% (37–39). Our study yielded comparable results, with incidence rates of 22.8% and 23.6% in the ETAA and OT groups, respectively. To prevent postoperative hypoparathyroidism, it is crucial to preserve the parathyroid glands *in situ* and ensure adequate blood supply, as hypovascularity is the primary underlying factor for transient hypoparathyroidism (40). The magnification provided by lumpectomy and the utilization of carbon nanoparticles can help preserve the parathyroid glands in situ (41). Generally, the superior parathyroid glands have a more consistent location, typically within 1.5 cm of the RLN entrance to the larynx, and are often surrounded by a small amount of adipose tissue, facilitating their preservation *in situ* during lateral lobe dissection (42). Conversely, the location of the inferior parathyroid glands shows significant variability, and despite being preserved *in situ* during CLND, the blood supply is often compromised, requiring autologous transplantation to maintain proper function (42). In our institution, autologous transplantation is often used for parathyroid glands with inadequate blood supply or those isolated during surgery.

In the ETAA procedure, the visual field is observed from the bottom to the top, which may pose a challenge for CLND. Nearby tissues like the sternum and clavicle may obstruct lower lymph nodes, leading to a visual blind spot. Therefore, some physicians have expressed concerns regarding the alignment of this approach with the principles of radical tumor treatment. However, our study showed no statistically significant difference in the number of CLND or pathologically confirmed metastases between the ETAA and COT groups in the selected patients diagnosed with stage T1 PTC. These results suggest that the ETAA group achieved comparable CLND to the COT group, which is in line with previous reports on ETAA for PTMC (43). Moreover, to evaluate the applicability of ETAA for CLND, we utilized a four-step method that significantly decreased the chance of harm to the parathyroid glands and RLN.

According to the Chinese recommendations and consensus on thyroid cancer, it is suggested that all patients diagnosed with PTC should undergo unilateral and bilateral CLND (24). However, this differs from the approach recommended by the ATA guidelines regarding the extent of CLND (28). The number of dissected lymph nodes and the presence of metastatic lymph nodes are important indicators for postoperative treatment and follow-up. Especially for patients who have undergone total thyroidectomy, a post-surgery sTg level < 1 mg/L is a critical factor in evaluating the effectiveness of surgical intervention and predicting the patient’s prognosis (28). Our study found no statistically significant difference in sTg levels < 1mg/L between the two groups. Therefore, our results indicate that the ETAA technique is equivalent to the COT technique concerning tumour integrity.

According to the ATA guidelines, the identification of new lesions or invasion of other organs within one year after surgery is classified as tumour recurrence or metastasis (28). If feasible, patients in such cases should be evaluated for a possible second surgery or retreatment with iodine 131 radiotherapy. The risk of thyroid cancer recurrence varies depending on several factors, such as the type of tumor, stage, pathological characteristics, and treatment method. Based on the available literature, the recurrence rate of PTC after standardized combined treatment ranges from 10-30% (28). During the follow-up period of our study, only one patient exhibited lymph node involvement in the lateral cervical region in the COT group, who subsequently underwent a surgical to remove the affected lymph nodes. The recurrence rate observed in this study was lower than that reported in the literature, which may be due to the careful preoperative ultrasound evaluation performed on each patient. This study excluded patients with tumours larger than 2 cm, thyroid capsule invasion, or lateral cervical lymph node metastasis, which may have influenced the decrease in recurrence rate.

Neck scarring is a major concern for patients, especially those of Asian descent. Laparoscopic surgery, which has accelerated the development of laparoscopic techniques, offers significant cosmetic advantages over open surgery. Our study confirms previous research findings that patients who underwent ETAA experienced increased cosmetic satisfaction and improved quality of life. However, it must be acknowledged that remote access thyroidectomy may be associated with some unconventional complications (44). Based on our clinical experience, we have observed that a small number of patients in the ETAA group may experience postoperative numbness in the neck and chest, possibly due to scarring (45). Nonetheless, these sensory disturbances can be effectively alleviated by early initiation of functional exercises and implementing massage (46). Importantly, these sensory symptoms are unlikely to adversely affect patients’ long-term quality of life.

This study has several limitations that must be acknowledged. First, as a single-center retrospective study, it is susceptible to selection bias in surgical access, although this was partially mitigated by the use of PSM in the enrolled population. Furthermore, the inclusion of a relatively small sample size of 138 matched patients with a short follow-up period may undermine the credibility of the study’s long-term tumour outcomes. Therefore, we strongly advocate conducting multicenter prospective studies with larger patient cohorts and longer follow-up periods to confirm the safety, feasibility, and tumour outcomes of ETAA for stage T1 PTC.

## Conclusion

ETAA is a safe and feasible surgical procedure for stage T1 PTC, with results similar COT and excellent cosmetic effects. However, it has a longer operative time and more drainage volume.

## Data availability statement

The original contributions presented in the study are included in the article/**Supplementary Material**. Further inquiries can be directed to the corresponding authors.

## Ethics statement

The studies involving humans were approved by The Ethics Committee of Zunyi Medical University Affiliated Hospital. The studies were conducted in accordance with the local legislation and institutional requirements. Written informed consent for participation was not required from the participants or the participants’ legal guardians/next of kin in accordance with the national legislation and institutional requirements. Written informed consent was obtained from the individual(s) for the publication of any potentially identifiable images or data included in this article.

## Author contributions

JH and DO contributed to the study concept and design, acquisition, analysis, and drafting of the article. YX and JY contributed to the interpretation of data. YG and XH contributed to data collection and article review. RQ and LZ supervised the study. All authors contributed to the article and approved the submitted version.
